# Predicting Outcomes of Hormone and Chemotherapy in the Molecular Taxonomy of Breast Cancer International Consortium (METABRIC) Study by Biochemically-inspired Machine Learning

**DOI:** 10.12688/f1000research.9417.3

**Published:** 2017-05-12

**Authors:** Eliseos J. Mucaki, Katherina Baranova, Huy Q. Pham, Iman Rezaeian, Dimo Angelov, Alioune Ngom, Luis Rueda, Peter K. Rogan

**Affiliations:** 1 Deparment of Biochemistry , University of Western Ontario, London, Canada; 2 School of Computer Science, University of Windsor, Windsor, Canada; 3 Department of Computer Science, University of Western Ontario, London, Canada; 4 CytoGnomix Inc, London, Canada

**Keywords:** Gene expression signatures, breast cancer, chemotherapy resistance, hormone therapy, machine learning, support vector machine, random forest

## Abstract

Genomic aberrations and gene expression-defined subtypes in the large METABRIC patient cohort have been used to stratify and predict survival. The present study used normalized gene expression signatures of paclitaxel drug response to predict outcome for different survival times in METABRIC patients receiving hormone (HT) and, in some cases, chemotherapy (CT) agents. This machine learning method, which distinguishes sensitivity vs. resistance in breast cancer cell lines and validates predictions in patients; was also used to derive gene signatures of other HT  (tamoxifen) and CT agents (methotrexate, epirubicin, doxorubicin, and 5-fluorouracil) used in METABRIC. Paclitaxel gene signatures exhibited the best performance, however the other agents also predicted survival with acceptable accuracies. A support vector machine (SVM) model of paclitaxel response containing genes *ABCB1, ABCB11, ABCC1, ABCC10, BAD, BBC3, BCL2, BCL2L1, BMF, CYP2C8, CYP3A4, MAP2, MAP4, MAPT, NR1I2, SLCO1B3, TUBB1, TUBB4A, *and *TUBB4B* was 78.6% accurate in predicting survival of 84 patients treated with both HT and CT (median survival ≥ 4.4 yr). Accuracy was lower (73.4%) in 304 untreated patients. The performance of other machine learning approaches was also evaluated at different survival thresholds. Minimum redundancy maximum relevance feature selection of a paclitaxel-based SVM classifier based on expression of genes *BCL2L1, BBC3, FGF2, FN1, *and *TWIST1* **was 81.1% accurate in 53 CT patients. In addition, a random forest (RF) classifier using a gene signature ( *ABCB1, ABCB11, ABCC1, ABCC10, BAD, BBC3, BCL2, BCL2L1, BMF, CYP2C8, CYP3A4, MAP2, MAP4, MAPT, NR1I2,SLCO1B3, TUBB1, TUBB4A, *and *TUBB4B*) predicted >3-year survival with 85.5% accuracy in 420 HT patients. A similar RF gene signature showed 82.7% accuracy in 504 patients treated with CT and/or HT. These results suggest that tumor gene expression signatures refined by machine learning techniques can be useful for predicting survival after drug therapies.

## Introduction

Current pharmacogenetic analysis of chemotherapy makes qualitative decisions about drug efficacy in patients (determination of good, intermediate or poor metabolizer phenotypes) based on variants present in genes involved in the transport, biotransformation, or disposition of a drug. We have applied a supervised machine learning (ML) approach to derive accurate gene signatures, based on the biochemically-guided response to chemotherapies with breast cancer cell lines ^1^, which show variable responses to growth inhibition by paclitaxel and gemcitabine therapies ^2, 3^. We analyzed stable ^4^ and linked unstable genes in pathways that determine their disposition. This involved investigating the correspondence between 50% growth inhibitory concentrations (GI _50_) of paclitaxel and gemcitabine and gene copy number, mutation, and expression first in breast cancer cell lines and then in patients ^1^. Genes encoding direct targets of these drugs, metabolizing enzymes, transporters, and those previously associated with chemo-resistance to paclitaxel (n=31 genes) were then pruned by multiple factor analysis (MFA), which indicated that expression levels of genes *ABCC10*, *BCL2*, *BCL2L1*, *BIRC5*, *BMF*, *FGF2*, *FN1*, *MAP4*, *MAPT*, *NKFB2*, *SLCO1B3*, *TLR6*, *TMEM243*, *TWIST1*, and *CSAG2* could predict sensitivity in breast cancer cell lines with 84% accuracy. The cell line-based paclitaxel-gene signature predicted sensitivity in 84% of patients with no or minimal residual disease (n=56; data from 5). The present study derives related gene signatures with ML approaches that predict outcome of hormone- and chemotherapies in the large METABRIC breast cancer cohort ^6^.

## Methods

**Figure 1.**
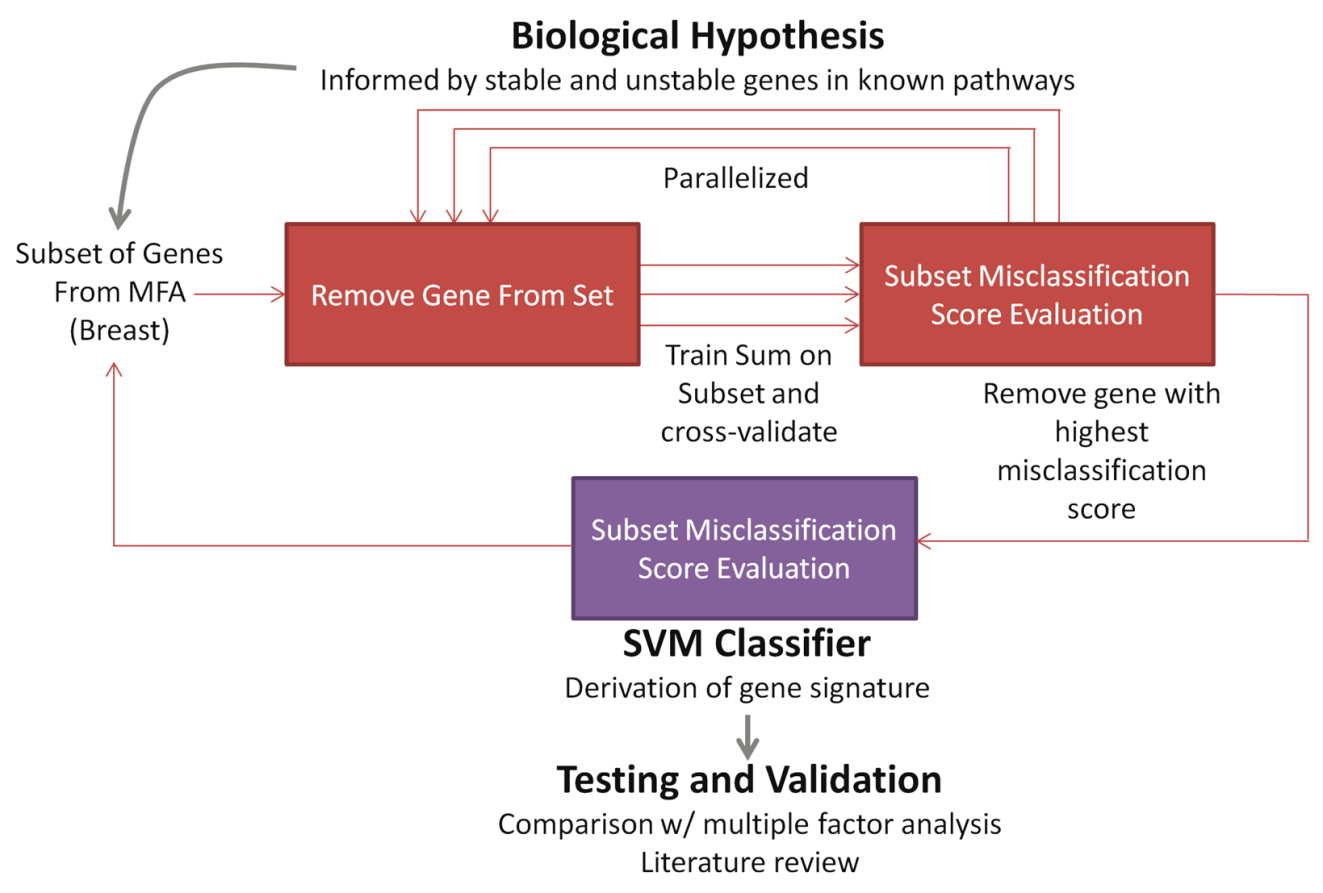
Biochemically-inspired SVM gene signature derivation workflow. The initial set of genes is carefully selected through the understanding of the drug and the pathways associated with it. A multiple factor analysis of the GI _50_ values of a training set of breast cancer cell lines and the corresponding expression levels of each gene in the initial set reduces the list of genes.

*SVM (Support Vector Machine) learning*: Previously, paclitaxel-related response genes were identified from peer-reviewed literature, and their expression and copy number in breast cancer cell lines were analyzed by multiple factor analysis of GI _50_ values of these lines ^2^ ( Figure 1). Given the expression levels of each gene, a SVM is evaluated on patients by classifying those with shorter survival time as resistant and longer survival as sensitive to hormone and/or chemotherapy using paclitaxel, tamoxifen, methotrexate, 5-fluorouracil, epirubicin, and doxorubicin. The SVM was trained using the function *fitcsvm* in MATLAB R2014a ^7^ and tested with either leave-one-out or 9 fold cross-validation (indicated in Table 1). The Gaussian kernel was used for this study, unlike Dorman *et al.* ^1^ which used the linear kernel. The SVM requires selection of two different parameters, C (misclassification cost) and sigma (which controls the flexibility and smoothness of Gaussians) ^8^; these parameters determine how strictly the SVM learns the training set, and hence if not selected properly, can lead to overfitting. A grid search evaluates a wide range of combinations of these values by parallelization. A Gaussian kernel selects the C and sigma combination that lead to the lowest cross-validation misclassification rate. A backwards feature selection (greedy) algorithm was designed and implemented in MATLAB in which one gene of the set is left out in a reduced gene set and the classification is then assessed; genes that maintain or lower the misclassification rate are kept in the signature. The procedure is repeated until the subset with the lowest misclassification rate is selected as the optimal subset of genes. These SVMs were then assessed for their ability to predict patient outcomes based on available metadata (see Figure 1 and reference 1). Interactive prediction using normalized expression values as input is available at http://chemotherapy.cytognomix.com.

**Table 1.** SVM gene expression signature performance on METABRIC patients.

Patient treatment	# of patients	Agent: *final gene* *signature (C* *and sigma)*	Accuracy (%)	Precision	F-Measure	MCC ^1^	AUC ^2^
Both CT and HT ^3^	84	Paclitaxel: *ABCC1, ABCC10, BAD,* *BIRC5, FN1, GBP1, MAPT, SLCO1B3,* *TMEM243, TUBB3, TUBB4B* *(C=10000, σ=10)*	78.6	0.787	0.782	0.559	0.814
		Tamoxifen: *ABCC2, ALB, CCNA2,* *E2F7, FLAD1, FMO1, NCOA2, NR1I2,* *PIAS4, SULT1E1 (C=100000, σ=100)*	76.2	0.761	0.760	0.510	0.701
		Methotrexate: *ABCC2, ABCG2,* *CDK2, DHFRL1 (C=10, σ=1)*	71.4	0.712	0.711	0.410	0.766
		Epirubicin *: ABCB1, CDA, CYP1B1,* *ERBB3, ERCC1, MTHFR, PON1,* *SEMA4D, TFDP2 (C=1000, σ=10)*	72.6	0.725	0.723	0.434	0.686
		Doxorubicin: *ABCC2, ABCD3, CBR1,* *FTH1, GPX1, NCF4, RAC2, TXNRD1* *(C=100000, σ=100)*	75.0	0.749	0.750	0.488	0.701
		5-Fluorouracil: *ABCB1, ABCC3,* *MTHFR, TP53 (C=10000, σ=100)*	71.4	0.714	0.714	0.417	0.718
CT and/or HT ^3, 4, 5, 6^	735	Paclitaxel: *BAD, BCAP29, BCL2,* *BMF, CNGA3, CYP2C8, CYP3A4,* *FGF2, FN1, NFKB2, NR1I2, OPRK1,* *SLCO1B3, TLR6, TUBB1, TUBB3,* *TUBB4A, TUBB4B, TWIST1* *(C=10000, σ=100)*	66.1	0.652	0.643	0.287	0.660
Deceased only ^2, 6, 7^ (CT and/or HT)	327	Paclitaxel: *ABCB11, BAD, BBC3,* *BCL2, BCL2L1, BIRC5, CYP2C8,* *FGF2, FN1, GBP1, MAPT, NFKB2,* *OPRK1, SLCO1B3, TMEM243* *(C=100, σ=10)*	75.3	0.752	0.752	0.505	0.763
No treatment ^3^	304	Paclitaxel: *ABCB1, ABCB11, BBC3,* *BCL2L1, BMF, CYP3A4, FGF2,* *GBP1, MAP4, MAPT, NR1I2, OPRK1,* *SLCO1B3, TUBB4A, TUBB4B,* *TWIST2 (C=100, σ=10)*	73.4	0.734	0.733	0.467	0.769

Initial gene sets preceding feature selection: Paclitaxel - *ABCB1, ABCB11, ABCC1, ABCC10, BAD, BBC3, BCAP29, BCL2, BCL2L1, BIRC5, BMF, CNGA3, CYP2C8, CYP3A4, FGF2, FN1, GBP1, MAP2, MAP4, MAPT, NFKB2, NR1I2, OPRK1, SLCO1B3, TLR6, TUBB1, TWIST1.* Tamoxifen - *ABCB1, ABCC2, ALB, C10ORF11, CCNA2, CYP3A4, E2F7, F5, FLAD1, FMO1, IGF1, IGFBP3, IRS2, NCOA2, NR1H4, NR1I2, PIAS4, PPARA, PROC, RXRA, SMARCD3, SULT1B1, SULT1E1, SULT2A1.* Methotrexate - *ABCB1, ABCC2, ABCG2, CDK18, CDK2, CDK6, CDK8, CENPA, DHFRL1*. Epirubicin - *ABCB1, CDA, CYP1B1, ERBB3, ERCC1, GSTP1, MTHFR, NOS3, ODC1, PON1, RAD50, SEMA4D, TFDP2*. Doxorubicin - *ABCB1, ABCC2, ABCD3, AKR1B1, AKR1C1, CBR1, CYBA, FTH1, FTL, GPX1, MT2A, NCF4, RAC2, SLC22A16, TXNRD1.* 5-Fluorouracil - *ABCB1, ABCC3, CFLAR, IL6, MTHFR, TP53, UCK2.* ^1^MCC: Matthews Correlation Coefficient. ^2^AUC: Area under receiver operating curve. ^3^ Surviving patients; ^4^ Analysis included patients in the METABRIC ‘discovery’ dataset only; ^5^ SVMs tested with 9 fold cross-validation, all others tested with leave-one-out cross-validation; ^6^ Includes all patients treated with HT,CT, combination CT/HT, either with or without combination radiotherapy; ^7^ Median time after treatment until death (> 4.4 years) was used to distinguish favorable outcome, ie. sensitivity to therapy.

**Figure 2.**
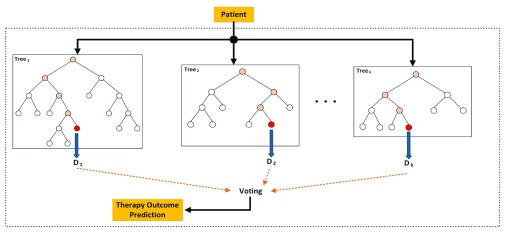
RF decision tree diagram depicts the therapy outcome prediction process of a given patient, using a RF consisting of *k* decision trees. Several DTs are built using different subsets of paclitaxel-related genes. The process starts from the root of each tree and if the expression of the gene corresponding to that node is greater than a specific value, the process continues through the right branch, otherwise it continues through the left branch until it reaches a leaf node; that leaf represents the prediction of the tree for that specific input. The decisions of all trees are considered and the one with the largest number of votes is selected as the patient outcome.

*RF (Random Forest) learning*: RF was trained using the WEKA 3.7 ^9^ data mining tool. This classifier uses multiple random trees for classification, which are combined via a voting scheme to make a decision on the given input gene set. A grid search was used to optimize the maximum number of randomly selected genes for each tree in RF, where k (maximum number of selected genes for each tree) was set from 1 to 19. Figure 2 depicts the therapy outcome prediction process of a given patient using a RF consisting of a series of decision trees derived from different subsets of paclitaxel-related genes.

*Augmented Gene Selection*: The most relevant genes (features) for therapy outcome prediction were found using the Minimum Redundancy and Maximum Relevance (mRMR) approach ^10^. mRMR is a wrapper approach that incrementally selects genes by maximizing the average mutual information between gene expression features and classes, while minimizing their redundancies:

$$mRMR=\underset{s}{\text{max}}\left[\frac{1}{\left|s\right|}\sum_{f_{i}\in S}I(f_{i},C)-\frac{1}{{\left|s\right|}^{2}}\sum_{f_{i},f_{j}\in S}I(f_{i},f_{j})\right]$$

where *f _i_* corresponds to a feature in gene set *S, I(f _i_,C)* is the mutual information between *f _i_* and class *C*, and *I(f _i_,f _j_)* is the mutual information between features *f _i_* and *f _j_*.

For this experiment, we used a 26-gene signature (genes *ABCB1, ABCB11, ABCC1, ABCC10, BAD, BBC3, BCL2, BCL2L1, BMF, CYP2C8, CYP3A4, MAP2, MAP4, MAPT, NR1I2, SLCO1B3, TUBB1, TUBB4A, TUBB4B, FGF2, FN1, GBP1, NFKB2, OPRK1, TLR6,* and *TWIST1*) as the base feature set. These genes were selected (in Dorman *et al.* ^1^) based either on their known involvement in paclitaxel metabolism, or evidence that their expression levels and/or copy numbers correlate with paclitaxel GI _50_ values. mRMR and SVM were combined to obtain a subset of genes that can accurately predict patient survival outcomes; here, we considered 3, 4 and 5 years as survival thresholds for breast cancer patients.

Performance was evaluated with several metrics. WEKA determined accuracy (ACC), the weighted average of precision and F-measure, the Matthews Correlation Coefficient (MCC) and the area under ROC curve (AUC).

## Results and discussion

Predicted treatment response for each individual METABRIC patient ^11^ The predicted and expected response to treatment for each individual METABRIC patient for each analyses listed in Table 1, Table 2 and Table 3 are indexed. Patients sensitive to treatment are labeled with ‘0’ while resistant patients are labeled ‘1’. Click here for additional data file. Copyright: © 2017 Mucaki EJ et al. 2017 Data associated with the article are available under the terms of the Creative Commons Zero "No rights reserved" data waiver (CC0 1.0 Public domain dedication).

**Table 2.** Results of applying RF to predict outcome of paclitaxel therapy.

Type of treatment	Survival years (as threshold)	# Patients	*K* (number of genes to be used in random selection)	Accuracy (True Positive - TP) (%)	Precision	F-Measure	MCC ^1^	AUC ^2^
Chemotherapy (CT)	3	53	7	56.6	0.510	0.524	-0.095	0.441
	4		7	69.8	0.698	0.698	0.396	0.700
	5		19	66.0	0.645	0.636	0.230	0.653
Hormone therapy (HT)	3	420	19	85.5	0.731	0.788	0.000	0.606
	4		9	78.6	0.715	0.706	0.069	0.559
	5		9	71.0	0.634	0.627	0.059	0.632
CT and/or HT	3	504	9	82.7	0.685	0.749	0.000	0.506
	4		19	73.6	0.647	0.648	0.039	0.527
	5		7	65.3	0.602	0.593	0.086	0.588

^1^MCC: Matthews Correlation Coefficient. ^2^AUC: Area under receiver operating curve; both Discovery and Validation patient datasets analyzed. RF predictions done using a gene panel consisting of 19 genes ( *ABCB1*, *ABCB11*, *ABCC1*, *ABCC10*, *BAD*, *BBC3*, *BCL2*, *BCL2L1*, *BMF*, *CYP2C8*, *CYP3A4*, *MAP2*, *MAP4*, *MAPT*, *NR1I2*, *SLCO1B3*, *TUBB1*, *TUBB4A*, *TUBB4B*).

**Table 3.** Results of mRMR feature selection for an SVM for predicting outcome of paclitaxel therapy.

**Data**	CT ^1^			HT			CT+HT		
**Survival years** **(as threshold)**	3	4	5	3	4	5	3	4	5
**# patients ^2^**	53			420			504		
**Accuracy (TP)** **(%)**	81.1	81.1	84.9	85.7	79.5	72.9	83.1	74.8	67.9
**Precision**	0.809	0.813	0.852	0.878	0.765	0.692	0.795	0.703	0.662
**F-Measure**	0.809	0.811	0.845	0.794	0.726	0.663	0.772	0.672	0.666
**MCC**	0.582	0.625	0.675	0.119	0.17	0.173	0.161	0.137	0.238
**AUC**	0.783	0.812	0.82	0.508	0.533	0.548	0.53	0.531	0.61
**SVM Par.** **(gamma)**	0.0	0.5	1.0	1.0	0.75	1.5	0.75	0.5	1.0
**SVM Par.** **(cost)**	64	128	8	2	64	2	16	2	2
**Selected** **genes**	*MAP4,* *GBP1*, *FN1*, *MAPT*, *BBC3*, *FGF2*, *NFKB2*, *TUBB4B*	*TWIST1*, *FN1*, *BBC3*, *FGF2*, *BCL2L1*	*ABCB11*, *BCL2*, *GBP1*, *SLCO1B3*, *ABCB1*, *BAD*, *TUBB4A*, *MAPT*, *NFKB2*, *TUBB4B*	*ABCB11*, *BCL2*, *MAP4*, *TUBB1*, *GBP1*, *SLCO1B3*, *ABCB1*, *BAD*, *TWIST1*, *FN1*, *TUBB4A*, *MAPT*, *OPRK1*, *BBC3*, *FGF2*, *NFKB2*, *ABCC1*, *NR1I2*	*BAD*, *GBP1*, *MAPT*, *BBC3*	*ABCB11*, *MAP4*, *SLCO1B3*, *BAD*, *FN1*, *OPRK1*, *BBC3*, *NFKB2*, *NR1I2*, *TUBB4B*	*ABCB11*, *SLCO1B3*, *BAD*, *TUBB4A*, *MAPT*, *BBC3*, *FGF2*, *NFKB2*, *ABCC1*, *NR1I2*	*ABCB11*, *BMF*, *BCL2*, *MAP4*, *TUBB1*, *GBP1*, *SLCO1B3*, *ABCB1*, *BAD*, *TWIST1*, *FN1*, *MAPT*, *OPRK1*, *BBC3*, *FGF2*, *NFKB2*, *ABCC1*, *NR1I2*, *TUBB4B*	*MAP4*, *GBP1*, *SLCO1B3*, *BAD*, *MAPT*, *OPRK1*, *BBC3*, *NFKB2*, *ABCC1*, *NR1I2*, *TUBB4B*

^1^For patients treated with CT with ≥4 Yr survival and CT+ HT for ≥ 5 Yr, the cost for the mRMR model was set to 64. Of those treated with CT for ≥ 4 Yr, genes were selected using a greedy, stepwise forward search, while in other cases, greedy stepwise backward search was used. Also, gamma = 0 in all cases. ^2^Predicted responses for individual METABRIC patients are provided in Dataset 1.

The performances of several ML techniques have been compared such that they distinguish paclitaxel sensitivity and resistance in METABRIC patients using its tumour gene expression datasets. We used mRMR to generate gene signatures and determine which genes are important for treatment response in METABRIC patients. The paclitaxel models are more accurate for prediction of outcomes in patients receiving HT and/or CT compared to other patient groups.

**Figure 3.**
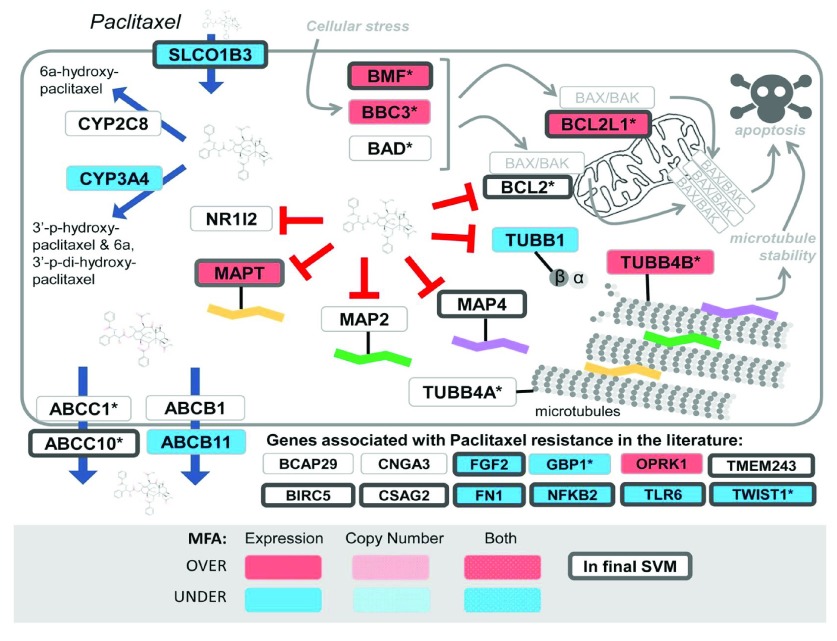
Schematic elements of gene expression changes associated with response to paclitaxel. Red boxes indicate genes with a positive correlation between gene expression or copy number, and resistance using multiple factor analysis. Blue demonstrates a negative correlation. Genes outlined in dark grey are those in a previously published paclitaxel SVM model (reproduced from reference 1 with permission).

SVMs and RF were trained using expression of genes associated with paclitaxel response, mechanism of action and stable genes in the biological pathways of these targets ( Figure 3). Pair-wise comparisons of these genes with those from MammaPrint and Oncotype Dx (other genomic classifiers for breast cancer) find that these signatures are nearly independent of each other, with only a single gene overlap. The distinct differences of these signatures are due to their methodology of derivation, based on different principles and for different purposes (i.e. drug response for a specific reagent). SVM models for drugs used to treat these patients were derived by backwards feature selection on patient subsets stratified by treatment or outcome ( Table 1). The highest SVM accuracy was found for the paclitaxel signature in patients treated with HT and/or adjuvant chemotherapy (78.6%). Since some CT patients were also treated with tamoxifen, methotraxate, epirubicin, doxorubicin and 5-fluorouracil, we also evaluated the performance of models developed for these drugs using the same algorithm. These gene signatures also had acceptable performance (accuracies between 71–76%; AUCs between 0.686 – 0.766). Leave-one-out validation (CT and HT, no treatment, and deceased patients) exhibited higher model performance than 9-fold crossvalidation (CT and/or HT, including patients treated with radiation).

The RF classifier was used to predict paclitaxel therapy outcome for patients that underwent CT and/or HT ( Table 2). The best performance achieved with RF showed an 85.5% overall accuracy using a 3-year survival threshold for distinguishing therapeutic resistance vs. sensitivity for those patients that underwent HT.

The best overall accuracy and AUC (sensitivity and specificity) for CT/HT patients using mRMR feature selection for SVM predicting outcome of paclitaxel therapy was obtained for CT patients with 4-year survival ( Table 3). Outcomes for HT patients with 3-year survival were predicted with 85.7% accuracy; however, the specificity was lower in this group. SVM combined with mRMR further improved accuracy of feature selection and prediction of response to hormone and/or chemotherapy based on survival time than either SVM or RF alone. Predicted treatment responses for individual METABRIC patients using the described ML techniques are indicated in Dataset 1.

Tumor co-variate information was provided by METABRIC, which included Estrogen receptors (ER), Progesterone Receptor (PR), HER2, Lymph Node (LN) and PAM50 subtypes. To assess model co-variate accuracy, predictions described in Table 1– Table 3 were broken down by subtype (available in Supplementary file 1). Subtypes with <20 individuals for a particular treatment combination were not analyzed. The deviation in classification accuracy between subtypes was mostly consistent with the average. One exception involved the RF and mRMR analyses, which was 8.3 to 23.0% below the average for (ER)-negative, (HER2)-positive and basal subtypes in patients treated with HT. However, this deviation was not observed for CT-treated patients with the (ER)-negative subtype, which was consistent with the fact that CT response was derived from the paclitaxel gene set. (ER)-negative patients primarily received CT ^6^. Further, the accuracy of the SVM models tested with CT and HT-treated patients was significantly higher for (HER2)-positive patients (26 correct, 3 misclassified; 90% accurate) compared to (HER2)-negative patients (40 correct, 15 misclassified; 73% accurate). *MAPT* expression (present in reduced ‘CT and HT’ paclitaxel model; Table 1) has been shown to segregate well with PAM50 luminal and basal subtypes1. When analyzing METABRIC patients, however, the accuracy of these two subtypes are nearly identical to the average (78.6%, where basal and luminal classification accuracy is 76.7% [n=30] and 76.2% [n=21], respectively).

We assessed the separate *Discovery* and *Validation* datasets, respectively, as training and test sets and repeated the previous experiments. In this scenario, the performance of the model was poor (slightly better than random). This occurred because the gene expression distributions of many of the paclitaxel-related genes in our signature were not reproducible between these two sets (based on Wilcoxon rank sum test, Kruskal-Wallis test and t-tests; Supplementary file 2). Cross-study validation allows for the comparison of classification accuracy between the generated gene signatures. The observed heterogeneity in gene expression highlights one of the many challenges of cross-validation of gene signatures between these data from the same study exhibit drastic differences (for example, *BCL2L1*; Supplementary file 2). Furthermore, these gene expression differences also affect the performance of these methods when these datasets were combined (compare Table 2 and Table 4 for RF; Table 3 and Table 5 for mRMR). We considered the possibility that the Discovery model might be subject to overfitting. We therefore performed cross-study validation of the Discovery set-signature with an independently-derived dataset (319 invasive breast cancer patients treated with paclitaxel and anthracycline chemotherapy ^5^). The mRMR+SVM CT-models performed well (4-year threshold model had an overall accuracy of 68.7%; 3-year threshold model exhibited lower overall accuracy [52%], but was significantly better at predicting patients in remission [74.2%]).

To evaluate the paclitaxel models without relying on the *Validation* dataset, the *Discovery* set was split into two distinct parts, consisting of 70% of the patient samples randomly selected for training, and a different set of 30% of samples for testing. This procedure was repeated 100 times using different combinations of training and test samples, and the median performance of these runs is reported ( Table 4 and Table 5). We also compared the performance of our mRMR+SVM model with the *K-TSP* model ^12^ ( Table 6). In most cases, our method outperformed K-TSP, based on its accuracy in classifying new patients. Starting with the same set of *Discovery* genes, we also trained a separate model using the *Validation* data, and tested this data by 70/30% cross-validation (accuracy for RF: 56–67% [CT], 67–83% [HT], 56–81% [CT-HT]; accuracy for mRMR: 33–56% [CT], 70–84% [HT], 64–82% [CT-HT]). In addition, we evaluated the performance of the model derived from the Discovery set on a different set of patients treated with paclitaxel ^5^. These results suggest that the aforementioned issue with *Discovery* training and *Validation* testing was primarily due to a batch effect, rather than to overfitting.

**Table 4.** Results of applying RF to predict outcome of the paclitaxel signature for the METABRIC *Discovery* patient set.

Type of treatment	Survival years (as threshold)	# Patients	*K* (number of genes to be used in random selection)	Accuracy (True Positive - TP) (%)	Precision	F-Measure	MCC	AUC
Chemotherapy (CT)	3	22	7	61.1	0.617	0.612	0.224	0.444
	4		7	66.7	0.643	0.646	0.189	0.715
	5		19	66.7	0.722	0.687	0.189	0.571
Hormone therapy (HT)	3	185	19	77.0	0.780	0.775	0.018	0.524
	4		9	79.1	0.733	0.710	0.084	0.527
	5		9	68.9	0.533	0.601	-0.133	0.594
CT and/or HT	3	221	9	80.2	0.677	0.734	-0.07	0.389
	4		19	54.8	0.554	0.551	-0.143	0.395
	5		7	60.5	0.567	0.579	0.016	0.479

Paclitaxel gene panel consisted of 19 genes ( *ABCB1*, *ABCB11*, *ABCC1*, *ABCC10*, *BAD*, *BBC3*, *BCL2*, *BCL2L1*, *BMF*, *CYP2C8*, *CYP3A4*, *MAP2*, *MAP4*, *MAPT*, *NR1I2*, *SLCO1B3*, *TUBB1*, *TUBB4A*, *TUBB4B*).

**Table 5.** Results of mRMR feature selection for an SVM for predicting outcome of the paclitaxel signature for the METABRIC *Discovery* patient set.

Treatment	CT ^1^			HT			CT+HT		
**Survival** **years (as** **threshold)**	3	4	5	3	4	5	3	4	5
**# patients**	22			185			221		
**Accuracy** **(TP) (%)**	57.14	57.14	85.7	81.8	70.9	63.6	71.2	69.7	71.2
**Precision**	0.595	0.686	0.735	0.726	0.670	0.532	0.647	0.629	0.693
**F-Measure**	0.571	0.623	0.791	0.769	0.686	0.562	0.668	0.628	0.666
**MCC**	0.167	-0.258	0.000	-0.080	0.032	-0.075	0.035	0.071	0.245
**AUC**	0.583	0.333	0.500	0.479	0.514	0.477	0.513	0.521	0.586
**SVM Par.** **(gamma)**	0.0	0.5	1.0	1.0	0.75	1.5	0.75	0.5	1.0
**SVM Par.** **(cost)**	64	128	8	2	64	2	16	2	2
**Selected** **genes**	*TWIST1* *BMF* *CYP2C8* *CYP3A4* *BCL2L1* *BBC3* *BAD* *MAP2* *MAPT* *NFKB2* *FN1*	*BCL2* *BMF* *CYP2C8* *CYP3A4* *BAD* *ABCC10* *NFKB2*	*MAP2* *BCL2* *BCL2L1* *BBC3* *MAPT* *GBP1* *NFKB2*	*TWIST1* *BCL2* *BMF* *CYP2C8* *CYP3A4* *BCL2L1* *BBC3* *TLR6* *BAD* *ABCB11* *ABCC1* *ABCC10* *MAP4* *MAPT* *NR1I2* *GBP1* *NFKB2* *OPRK1* *FN1*	*TWIST1* *CYP2C8* *CYP3A4* *BCL2L1* *BBC3* *TLR6* *ABCB11* *ABCC1* *ABCC10* *MAP2* *MAPT* *NR1I2* *GBP1* *NFKB2* *FN1*	*TWIST1* *BMF* *CYP2C8* *CYP3A4* *BCL2L1* *BBC3* *ABCB11* *ABCC1* *ABCC10* *MAP2* *MAP4* *MAPT* *NR1I2* *GBP1* *NFKB2* *OPRK1*	*BMF* *CYP2C8* *BCL2L1* *BBC3* *BAD* *ABCC1* *ABCC10* *MAP4* *NR1I2* *GBP1* *NFKB2* *OPRK1* *FN1*	*TWIST1* *BMF* *CYP2C8* *CYP3A4* *BCL2L1* *BBC3* *TLR6* *ABCB11* *ABCC1* *ABCC10* *MAP2* *MAP4* *MAPT* *NR1I2* *GBP1* *NFKB2* *OPRK1* *FN1*	*TWIST1* *BMF* *CYP3A4* *BCL2L1* *BBC3* *TLR6* *BAD* *ABCB11* *ABCC1* *MAP2* *MAP4* *MAPT* *NR1I2* *GBP1* *NFKB2* *OPRK1* *FN1*

^1^For patients treated with CT with ≥4 Yr survival and CT+ HT for ≥ 5 Yr *,* the cost for the mRMR model was set to 64. Of those treated with CT for ≥ 4 Yr, genes were selected using a greedy, stepwise forward search, while in other cases, greedy stepwise backward search was used. Also, gamma = 0 in all cases.

**Table 6.** Comparison between our mRMR+SVM method and K-TSP method on *Discovery* patient set of the METABRIC data.

Data	CT			HT			CT+HT		
**Survival years**	3	4	5	3	4	5	3	4	5
**# patients**	22			185			221		
**mRMR+SVM Accuracy (%)**	57.14	57.14	85.7	81.8	70.9	63.6	71.21	69.70	71.21
**K-TSP** ^12^ **Accuracy (%)**	57.14	28.57	28.57	80.91	68.18	69.19	71.21	54.55	53.03

The performances of several ML techniques have been compared such that they distinguish paclitaxel sensitivity and resistance in METABRIC patients using its tumour gene expression datasets. We used mRMR to generate gene signatures and determine which genes are important for treatment response in METABRIC patients. The paclitaxel models are more accurate for prediction of outcomes in patients receiving HT and/or CT compared to other patient groups.

While not a replication study *sensu stricto*, the initial paclitaxel gene set used for feature selection was the same as in our previous study ^1^. Predictions for the METABRIC patient cohort, which was independent of the previous validation set ^5^ used in Dorman *et al.* ^1^, of the either same (SVM) or different ML methods (RF and SVM with mRMR) exhibited comparable or better accuracies than our previous gene signature ^1^.

These techniques are powerful tools which can be used to identify genes that may be involved in drug resistance, as well as predict patient survival after treatment. Future efforts to expand these models to other drugs may assist in suggesting preferred treatments in specific patients, with the potential impact of improving efficacy and reducing duration of therapy.

## Conclusion

In this study we used METABRIC dataset to predict outcome for different survival times in patients receiving hormone (HT) and, in some cases, chemotherapy (CT) agents. We used published literature and various machine learning methods in order to identify optimal subsets of genes from a biologically-relevant initial gene set that can accurately predict therapeutic response of patients who have received chemotherapy, hormone therapy or a combination of both treatments. The SVM methodology has been previously shown to outperform randomized gene sets ^1^. The predictions made by our method are based on the level of an individual drug. Genomic information has been shown to correlate with tumor therapy response in previous studies ^5, 13– 17^. From these studies, analytical methods have been used to develop gene signatures for chemotherapy resistance prediction ^5^, subtypes (PAM50), and metastatic risk stratification (Oncotype DX™, MammaPrint ^®)^. We also examined the method exhibiting the best performance in the Sage Bionetworks / DREAM Breast Cancer Prognosis Challenge ^18^, which was also phenotype-based, however it produces outcome signatures based on molecular processes rather than the cancer drugs themselves. While interesting and informative, the results cannot be directly compared. Our approach may be useful for selecting specific therapies in patients that would be expected to produce a favorable response.

## Data availability

The data referenced by this article are under copyright with the following copyright statement: Copyright: © 2017 Mucaki EJ et al.

Data associated with the article are available under the terms of the Creative Commons Zero "No rights reserved" data waiver (CC0 1.0 Public domain dedication). 

*Patient data*: The METABRIC datasets are accessible from the European Genome-Phenome Archive (EGA) using the accession number EGAS00000000083 ( https://www.ebi.ac.uk/ega/studies/EGAS00000000083). Normalized patient expression data for the *Discovery* (EGAD00010000210) and *Validation* sets (EGAD00010000211) were retrieved with permission from EGA. Corresponding clinical data was obtained from the literature ^6^. While not individually curated, HT patients were treated with tamoxifen and/or aromatase inhibitors, while CT patients were most commonly treated with cyclophosphamide-methotrexate-fluorouracil (CMF), epirubicin-CMF, or doxorubicin-cyclophosphamide.

F1000Research: Dataset 1. Predicted treatment response for each individual METABRIC patient, 10.5256/f1000research.9417.d149864 ^11^

## Supplementary material

**Supplementary File 1: Accuracy of SVM, RF and mRMR by Patient Co-variates.**

Accuracy of all models described in Table 1– Table 3 were further broken down by provided patient subtype information (ER, HER, PR, PAM50, and LN).

.

**Supplementary File 2: Variation of Gene Expression Distribution between Discovery and Validation Datasets.**

Whisker plots showing the distribution of expression in the *Discovery* and *Validation* METABRIC datasets for 26 genes used in the paclitaxel gene signature.
