# Supplementary material for: Predicting Outcomes of Hormone and Chemotherapy in the Molecular Taxonomy of Breast Cancer International Consortium (METABRIC) Study by Biochemically-inspired Machine Learning
Source: F1000Res. 2017 May 12;5:2124. Originally published 2016 Aug 31. [Version 3] doi: 10.12688/f1000research.9417.3 (PMC5461908; doi:10.12688/f1000research.9417.3)
Supplement: Peer review history

# Peer review: Referee response for version 3

**DOI:** 10.5256/f1000research.12412.r22653

**Published:** 2017-05-31

**Authors:** Elana Judith Fertig

**Affiliations:** 1 Division of Oncology Biostatistics and Bioinformatics, School of Medicine, Johns Hopkins University, Baltimore, MD, USA

The authors have addressed all previous comments.

I have read this submission. I believe that I have an appropriate level of expertise to confirm that it is of an acceptable scientific standard.

# Peer review: Referee response for version 2

**DOI:** 10.5256/f1000research.11525.r19726

**Published:** 2017-02-21

**Authors:** Chun-Wei Tung

**Affiliations:** 1 School of Pharmacy, Kaohsiung Medical University, Kaohsiung, Taiwan

The authors have addressed all the concerns raised from the previous review. A minor comment for the batch effects is given in the follows. As batch effects are expected for heterogeneous datasets, the direct application of prediction model built on the discovery dataset to the validation dataset would be incorrect and usually result in poor performance. To show the usefulness of the gene signatures while minimizing the batch effects, the authors might consider to run cross-validation on the validation dataset alone using the gene signatures obtained from discovery dataset.

I have read this submission. I believe that I have an appropriate level of expertise to confirm that it is of an acceptable scientific standard.

# Response

**Published:** 2017-04-21

**Authors:** Peter Rogan

**Affiliations:** University of Western Ontario, Canada

Thank you for your suggestion. As recommended, we have repeated the 70/30% cross-validation analysis performed in the manuscript (Tables 4 and 5) with the same genes obtained from the *Discovery* dataset (Tables 4 and 5), but using the *Validation* dataset alone for training and testing. We found that this analysis had a similar performance level as the analysis reported in the main manuscript (Tables 4 and 5). There are exceptions. The mRMR+SVM gene signature developed using “CT-only” patients at a 5-year threshold was much less accurate using the *Validation* data. However, the “CT-only” subset of the *Validation* dataset is small (N=31), and thus variability is not unexpected. Overall, this analysis suggests that the cross-validation issue was indeed mostly due to batch effects.

The following sentence was written in the main text which describes this result:

“Starting with the same set of *Discovery* genes, we also trained a separate model using the *Validation* data, and tested this data by 70/30% cross-validation  (accuracy for RF: 56-67% [CT], 67-83% [HT], 56-81% [CT-HT]; accuracy for mRMR: 33-56% [CT], 70-84% [HT], 64-82% [CT-HT]).”

# Peer review: Referee response for version 2

**DOI:** 10.5256/f1000research.11525.r19727

**Published:** 2017-02-03

**Authors:** Elana Judith Fertig

**Affiliations:** 1 Division of Oncology Biostatistics and Bioinformatics, School of Medicine, Johns Hopkins University, Baltimore, MD, USA

The authors were very responsive to the previous round of reviews, including more robust cross-validation and cross-study validation and comparison with other classifiers. Particular concerns remain that the author’s conclusions that it is inappropriate to perform cross-study validation due to batch effects are incorrect, particularly since this challenging task is essential to assess overfitting and for clinical translation of classifiers. In addition, the conclusion was insufficiently revised to place their classifier in the context of the broader literature in this field.

**Methods**

1. Abbreviations SVM and RF must be spelled out as Support Vector Machine and Random Forest on first use. This was not addressed in the revised methods section.

**Results**

1. The authors did perform a robust cross-study validation, as requested in the previous review. We agree this is challenging, due in part to batch effects as reported in this manuscript. However, such cross-study validation is essential to assess the accuracy of classifiers. It is also essential to have translation of genomic signatures into the clinic, where even different assays may be used. To address these concerns the authors must do the following: (a) Remove the sentence “This heterogeneity indicates that it is inappropriate to test our gene expression signatures derived by one of these datasets using the other dataset.” (b) Discuss the importance of cross-study validation, challenges in this application, and potential of overfitting of suggested by these results.
2. The author’s response that specific therapies were not provided in METABRIC is incorrect. According to Curtis *et al.*, (2012) “Nearly all oestrogen receptor (ER)-positive and/or lymph node (LN)-negative patients did not receive chemotherapy, whereas ER-negative and LN-positive patients did. Additionally, none of the HER2 ^+^ patients received trastuzumab. As such, the treatments were homogeneous with respect to clinically relevant groupings.” Therefore, the previous criticism #12 remains. Covariates such as ER/HER2/LN or PAM50 subtypes must be included in a table describing the sample cohorts remains. In addition, accuracy must be computed separately for these co-variates or included in the machine learning model.

**Conclusion**

1. The discussion is insufficient. It still lacks sufficient context of existing genomics classifiers in the literature. The discrepancy between their algorithm and clinical assays is confusing in revised sentence “Unlike Mammaprint and Oncotype Dx tests, this model focuses on predicting survival prediction based on gene expression in the tumor, presumably before or during drug therapy.” As written, it appears to disregard the long history of predicting clinical outcome from gene expression involved in developing these classifiers from gene expression data (e.g., van't Veer *et al.,* 2002) into clinical assays based upon expression of smaller numbers of genes.
2. Based on the previous review, the authors include context with other predictions of the METABRIC data in the response to the reviewers. This must also be included in the Conclusion to assess the relevance of their findings in the literature.

I have read this submission. I believe that I have an appropriate level of expertise to confirm that it is of an acceptable scientific standard, however I have significant reservations, as outlined above.

## References

[1] CurtisCShahSPChinSFTurashviliGRuedaOMDunningMJSpeedDLynchAGSamarajiwaSYuanYGräfSHaGHaffariGBashashatiARussellRMcKinneySMETABRIC GroupLangerødAGreenAProvenzanoEWishartGPinderSWatsonPMarkowetzFMurphyLEllisIPurushothamABørresen-DaleALBrentonJDTavaréSCaldasCAparicioS: The genomic and transcriptomic architecture of 2,000 breast tumours reveals novel subgroups. *Nature*.2012;486(7403) : 10.1038/nature10983 346-52 10.1038/nature10983 22522925PMC3440846

[2] van 't VeerLJDaiHvan de VijverMJHeYDHartAAMaoMPeterseHLvan der KooyKMartonMJWitteveenATSchreiberGJKerkhovenRMRobertsCLinsleyPSBernardsRFriendSH: Gene expression profiling predicts clinical outcome of breast cancer. *Nature*.2002;415(6871) : 10.1038/415530a 530-6 10.1038/415530a 11823860

# Response

**Published:** 2017-04-21

**Authors:** Peter Rogan

**Affiliations:** University of Western Ontario, Canada

**Methods**

*Comment 1. Abbreviations SVM and RF must be spelled out as Support Vector Machine and*

*Random Forest on first use. This was not addressed in the revised methods section.*

Response: These abbreviations are now spelled out upon their first use in the main text (Methods section).

**Results**

*Comment 2. The authors did perform a robust cross-study validation, as requested in the previous review. We agree this is challenging, due in part to batch effects as reported in this manuscript. However, such cross-study validation is essential to assess the accuracy of classifiers. It is also essential to have translation of genomic signatures into the clinic, where even different assays may be used. To address these concerns the authors must do the following: (a) Remove the sentence “This heterogeneity indicates that it is inappropriate to test our gene expression signatures derived by one of these datasets using the other dataset.” (b) Discuss the importance of cross-study validation, challenges in this application, and potential of overfitting of suggested by these results.*

Response: In regards to this point:

(a) This sentence has been removed, as requested.

(b) To address concerns regarding potential overfitting of our models, we cross-validate the acquired models to a non-METABRIC data set (from an independent study). In the Sage Bionetworks / DREAM Breast Cancer Prognosis Challenge, cross-study validation was performed using the “OsloVal” data set, which consists of gene expression and copy number data from 184 breast cancer patients (Margolin *et al.*, 2013). However, this dataset is not publically available and requires Ethics Board / IRB Review which we did not believe to be worth the effort. Instead, we performed cross-study validation on the gene expression of 310 breast cancer patients made publically available by Hatzis *et al.* (2011).

Analysis of this dataset was successful for the mRMR + SVM models developed using chemotherapy-treated patient (“CT” models), where the threshold for resistance was set to 3-years and 4-years. The “CT 3-year” model performed well predicting responsive patients (74.2% accuracy), while the “CT 4-year” model performed better predicting non-responsive patients (75.1% accuracy). The “CT 4-year” model outperformed the “CT 5-year” model for both sensitive and resistant patient data sets.

Random Forest and mRMR+SVM models which used hormone-treated patients (“HT” and “CT+HT”) were much less accurate compared to the “CT-only” models, and predict patients a large percentage of patients from the Hatzis data as sensitive.

In the main manuscript, we have replaced the removed sentence from (a) and have written the following:

“Cross-study validation allows for the comparison of classification accuracy between the generated gene signatures. The observed heterogeneity in gene expression highlights one of the many challenges of cross-validation of gene signatures between these  data from the same study exhibit drastic differences (for example, *BCL2L1*; Supplementary file 2). Furthermore, these gene expression differences also affect the performance of these methods when these datasets were combined (compare Table 2 and Table 4 for RF; Table 3 and Table 5 for mRMR). We considered the possibility that the Discovery model might be subject to overfitting. We therefore performed cross-study validation of the Discovery set-signature with an independently-derived dataset (319 invasive breast cancer patients treated with paclitaxel and anthracycline chemotherapy ^5^). The mRMR+SVM CT-models performed well (4-year threshold model had an overall accuracy of 68.7%; 3-year threshold model exhibited lower overall accuracy [52%], but was significantly better at predicting patients in remission [74.2%]).”

**References**

Margolin AA, *et al.* Systematic analysis of challenge-driven improvements in molecular prognostic models for breast cancer. Sci Transl Med. 2013 Apr 17;5(181):181re1. doi: 10.1126/scitranslmed.3006112.

Hatzis, C., *et al.* 2011. A genomic predictor of response and survival following taxane-anthracycline chemotherapy for invasive breast cancer. JAMA. 305, 1873–1881.

*Comment 3. The author’s response that specific therapies were not provided in METABRIC is incorrect. According to Curtis et al., (2012) “Nearly all oestrogen receptor (ER)-positive and/or lymph node (LN)-negative patients did not receive chemotherapy, whereas ER-negative and LN-positive patients did. Additionally, none of the HER2 ^+^ patients received trastuzumab. As such, the treatments were homogeneous with respect to clinically relevant groupings.” Therefore, the previous criticism #12 remains. Covariates such as ER/HER2/LN or PAM50 subtypes must be included in a table describing the sample cohorts remains. In addition, accuracy must be computed separately for these co-variates or included in the machine learning model.*

Response: Thank you for the clarification regarding patient treatment. As a response, we have added an additional supplementary table which breaks down the accuracy of our models by subtype (ER, HER2, PR, LN and PAM50; Dataset 2). In the main text, we note that accuracy of most models are consistent between subtypes (+/- 10% deviation in accuracy). Subtypes with less than twenty individuals were ignored due to its small sample size. The following deviations in accuracy were noted:

1. Random Forest and mRMR models are shown to be consistently more accurate in predicting ER+, HER2- when treated with hormone therapy (both “HT” and “CT and/or HT” categories), when compared to ER- and HER2+ patients. The PAM50 basal subtype is consistently low in accuracy when testing patients treated with hormone therapy. This is most likely partially influenced by the RF and mRMR models for ‘HT’ to more often predict patients as sensitive, combined with the fact that ER+ and HER2- patients were more likely to response to therapy. It is important to note that the accuracy of predictions by RF and mRMR with patients treated only with chemotherapy was fairly consistent across all available subtypes (+/- 10% accuracy).
2. SVM paclitaxel models performed significantly better with HER2+ patients (26 correct, 3 misclassified; 90% accurate) in HER2- patients (40 correct, 15 misclassified; 73% accurate) when tested on patients treated with both hormone and chemotherapy. In Dorman et al (2016), it was stated that *MAPT* expression (which is present in the paclitaxel model) segregated with PAM50 luminal and basal subtypes. For this model, the accuracies of these subtypes are nearly identical to the accuracy of the entire subset.

Text describing these results can be found in the third paragraph of the results.

**Conclusion**

*Comment 4. The discussion is insufficient. It still lacks sufficient context of existing genomics classifiers in the literature. The discrepancy between their algorithm and clinical assays is confusing in revised sentence:*

*“Unlike Mammaprint and Oncotype Dx tests, this model focuses on predicting survival prediction based on gene expression in the tumor, presumably before or during drug therapy.”*

*As written, it appears to disregard the long history of predicting clinical outcome from gene expression involved in developing these classifiers from gene expression data (e.g., van't Veer et al., 2002) into clinical assays based upon expression of smaller numbers of genes.*

Response: We have removed the indicated sentence, which we agree was insufficient to the comment from the previous iteration of this article: “Must be discussed in the context of existing genomics classifiers for breast cancer (e.g., OncotypeDx and/or Mammaprint)”.

We in no way meant to ignore the long history of predicting clinical outcome from gene expression (as well as other genomic factors). A discussion on this topic was not included in earlier submissions as it initially had an imposed word length limit (upon first submission). We did, however, reference other articles which do discuss this topic. In Dorman *et al.* (2016), which described some of the methodology for initial gene selection that this study was based on, these contributions  are  well-referenced, including the history of the prediction of clinical outcome from genomic status:

“Previous studies have derived associations between the genomic status of one or more genes and tumor response to certain therapies (Duan *et al.*, 2003; Glinsky *et al.*, 2005; Hatzis *et al.*, 2011; Ma *et al.*, 2004; Rajput *et al.*, 2013; van't Veer *et al.*, 2002).

Correlations between single gene expression and tumor resistance (Duan *et al.*, 2003, 1999) do not take into account multiple mechanisms of resistance or assess interactions between multiple genes. ABC transporter overexpression has long been shown to confer resistance, but enzymatic or functional inhibition has not substantially improve patient response to chemotherapy (Samuels *et al.*, 1997).

Multi-gene analytical approaches have previously been successful in deriving prognostic gene signatures for metastatic risk stratification (Oncotype DX™, MammaPrint ^®^), subtypes (PAM50), and efforts have been made to predict chemotherapy resistance (Hess *et al.*, 2006; Hatzis *et al.*, 2011). “

In response to Dr. Fertig’s comments, we have added a short discussion with citations of previously published approaches (including MammaPrint and Oncotype DX):

“Genomic information has been shown to correlate with tumor therapy response in previous studies ^5,12-16^. From these studies, analytical methods have been used to develop gene signatures for chemotherapy resistance prediction ^5^, subtypes (PAM50), and metastatic risk stratification (Oncotype DX™, MammaPrint ^®^).”

*Comment 5. Based on the previous review, the authors include context with other predictions of the METABRIC data in the response to the reviewers. This must also be included in the Conclusion to assess the relevance of their findings in the literature.*

Response: We have added the indicated text from the previous ‘response to the reviewers’ (modified) to the Conclusions:

“We also examined the method exhibiting the best performance in the Sage Bionetworks / DREAM Breast Cancer Prognosis Challenge ^17^, which was also phenotype-based, however it produces outcome signatures based on molecular processes, rather than the cancer drugs themselves. While interesting and informative, the results cannot be directly compared.”

Please note that the majority of entries in the DREAM project were not fully curated and only exist as source code. Analyzing these files to determine what methodology was attempted by these groups is beyond the scope of our study. A description of the second place of the METABRIC phase of the DREAM challenge is provided in the link below. This link describes how the METABRIC data is trained using a bipartite graphing as input for linear models, boost models, and RankSVM. While they state that RankSVM was the least successful between the three methods, it does not appear that this particular study has been published to the literature. As a result, we cannot fully review their results, and thus cannot be compared to our methodology in the main manuscript.

https://sagesynapse.wordpress.com/2012/11/01/breast-cancer-challenge-team-pitttransmed-places-second-for-metabric-phase-of-the-challenge/

# Peer review: Referee response for version 1

**DOI:** 10.5256/f1000research.10141.r16345

**Published:** 2016-10-03

**Authors:** Chun-Wei Tung

**Affiliations:** 1 School of Pharmacy, Kaohsiung Medical University, Kaohsiung, Taiwan

This study proposed prediction methods using SVM and RF classifiers with mRMR selected feature sets from cell line data and demonstrate its prediction ability for outcomes from METABRIC patient cohort. The classifiers with good prediction performance show the usefulness of combining domain knowledge with feature selection techniques. However, some details essential for reproducibility and interpretation are missing.

 Required information is listed in the following.

1. What are the values of parameters for SVM and RF classifiers and the methods for parameter selection (by default or other selection methods)?
2. The development and evaluation of models for patient data are not clear. Whether the models were trained using partial data from METABRIC or only leave-one-out cross-validation was applied? If cross-validation is the case, then what is the model offered at the online server because there will be more than one models created, and whether the cross-validation is involved in the feature selection process that often leads to an overestimation of the performance. For the case of training on partial data, both training and test performance are essential information for evaluating the robustness of models.
3. Since some of the datasets are highly imbalanced, the numbers of positives and negatives, as well as sensitivity and specificity are more important than accuracy for interpreting the results as a high accuracy with a low AUC could be the result of all positive/negative predictions on an imbalanced dataset. Listing all the information along with the accuracy and AUC will help the interpretation of prediction performances.  

I have read this submission. I believe that I have an appropriate level of expertise to confirm that it is of an acceptable scientific standard, however I have significant reservations, as outlined above.

# Response

**Published:** 2017-01-13

**Authors:** Peter Rogan

**Affiliations:** University of Western Ontario, Canada

*Comment 1:What are the values of parameters for SVM and RF classifiers and the methods for parameter selection (by default or other selection methods)?*

 Response: The parameter values for these classifiers have been added to the Tables 1-5.

 In regards to parameter selection, the first paragraph of the methods now describes C and Sigma selection as a grid search to find the values with the lowest cross-validation misclassification rate. Similarly for RF, a grid search was used to optimize the maximum number of randomly selected genes for each tree (second paragraph of Methods section).

*Comment 2: The development and evaluation of models for patient data are not clear. Whether the models were trained using partial data from METABRIC or only leave-one-out cross-validation was applied? If cross-validation is the case, then what is the model offered at the online server because there will be more than one models created, and whether the cross-validation is involved in the feature selection process that often leads to an overestimation of the performance. For the case of training on partial data, both training and test performance are essential information for evaluating the robustness of models.*

 Response: We obtained new results for both RF and mRMR+SVM models when we use discovery set as training set and validation set as test set, the performance of the model was poor. After more investigation we found that there happened to be a large variation between gene expression of 26 targeted genes between discovery and validation set (please see Supplementary Dataset 2). Hence, building any classifier using discovery and validation set as training and test set in their current forms will result of poor performance, since the training and test sets are vastly different.

 However, we did carry out another experiment on discovery set solely and used 70% of data for training and remaining 30% for test the performance of the model. The results have been added to the manuscript (Tables 4 and 5).

*Comment 3: Since some of the datasets are highly imbalanced, the numbers of positives and negatives, as well as sensitivity and specificity are more important than accuracy for interpreting the results as a high accuracy with a low AUC could be the result of all positive/negative predictions on an imbalanced dataset. Listing all the information along with the accuracy and AUC will help the interpretation of prediction performances.*

 Response: As previously mentioned, we have added more performance measures including MCC and AUC. They have been added Tables 1-5 of the manuscript.

# Peer review: Referee response for version 1

**DOI:** 10.5256/f1000research.10141.r16733

**Published:** 2016-09-30

**Authors:** Elana Judith Fertig

**Affiliations:** 1 Division of Oncology Biostatistics and Bioinformatics, School of Medicine, Johns Hopkins University, Baltimore, MD, USA

This study develops SVM and RF algorithms built upon previously learned gene signatures of therapeutic response to breast cancer. The algorithms are applied and compared to predict patient survival under different treatment conditions in METABRIC data. The analyses and comparisons are robust and this study provides a useful assessment of biologically-driven classifiers. The three major areas that require improvement before the article is indexed are as follows, and described in further detail below.

1. The methods require further clarification to distinguish differences between this study and the previous study as well as the parameters of the machine learning algorithms.
2. Accuracy in the results must better distinguish results on independent test and training sets.
3. Classifiers must be put in the context of other existing genomics classifiers used in breast cancer and/or previously published in Mammaprint data.

 

**Title and Abstract**

 Acceptable

**Article content**

***Methods***

1. Abbreviations SVM and RF must be spelled out as Support Vector Machine and Random Forrest on first use in Methods.
2. Writing in *SVM learning *subsection of *Methods* requires clarification to distinguish which of these methods were developed in the previous *Molecular Oncology* publication and which were developed as part of this publications.
3. Details about the SVM learning algorithm are included in the caption to Figure 1, but must also be included and completely described in text for the corresponding section of the methods.
4. No equations are provided to describe the role of the parameters C **and sigma. It is also unclear whether this greedy search is implemented by the Matlab function *fitcsvm *or uses custom code developed by the authors.

 

***Results***

1. Need to specify whether reported accuracies are computed with leave-one-out cross validation or 9-fold cross validation (described in Methods).
2. Ideally, given the size of METABRIC data they would be calculated on independent training (first 1000 patient samples) and training (last 1000 patient samples) datasets.
3. AUC must be computed separately for discovery and validation sets (Table 2).
4. It is unclear whether the previous validation set described in the sentence “Predictions for the METABRIC patient cohort, which was independent of the previous validation set” refers to a validation set used in this publication or the previous publication.
5. Covariates such as ER/PR or PAM50 subtypes must be included in a table describing the sample cohorts. Accuracy must be computed separately for these co-variates or they must also be included as co-variates in the machine-learning model.
6. Ideally accuracy would be compared to existing breast cancer classifiers (e.g., using code from Marchionni *et al.,* *BMC Genomics*, 2013) and/or survival curves reported in the literature.

 

**Conclusions**

1. Must be discussed in the context of existing genomics classifiers for breast cancer (e.g., OncotypeDx and/or Mammaprint).
2. Results must be put in context with other predictions on METABRIC data, e.g., outcomes from the DREAM contest.

 

***Data***

 Acceptable

I have read this submission. I believe that I have an appropriate level of expertise to confirm that it is of an acceptable scientific standard, however I have significant reservations, as outlined above.

# Response

**Published:** 2017-01-13

**Authors:** Peter Rogan

**Affiliations:** University of Western Ontario, Canada

*Comment 1: The methods require further clarification to distinguish differences between this study and the previous study as well as the parameters of the machine learning algorithms.*

 Response: The first paragraph of the Methods describes Support Vector Machine learning, which has been greatly expanded upon. Differences in SVM methodology between the two studies are indicated there (i.e. a Gaussian kernel was used instead of a linear kernel). All other feature selection methods described in the manuscript (Random Forest, mRMR) were not used in Dorman *et al.*, 2016.

 The parameters for machine learning algorithms have been incorporated in the manuscript, and can be found in the footnote section of each data table.

*Comment 2: Accuracy in the results must better distinguish results on independent test and training sets.*

 Response: The Validation dataset showed a distinct overall expression profile from the Discovery set, possibly due to batch effects, which are well known. We added another experiment to the manuscript by splitting the Discovery set into Training and Test sets. The model was trained using 70% of the data and then tested using the remaining 30% of data as test set. We repeated this procedure 100 times and took the median as the final performance result. The results are presented in Tables 4 and 5 of the manuscript.

*Comment 3: Classifiers must be put in the context of other existing genomics classifiers used in breast cancer and/or previously published in Mammaprint data.*

 Response: We have added two sentences in the second paragraph of the “Results and Discussion” section which describes the comparison of our gene signature to those from MammaPrint and Oncotype Dx. Pair-wise comparison of these three signatures show that they are nearly independent of one another.

**Methods**

*Comment 4: Abbreviations SVM and RF must be spelled out as Support Vector Machine and Random Forest on first use in Methods.*

 Response: We thank the reviewer for this suggestion. It has been addressed in the Methods section of the manuscript.

**

*Comment 5: Writing in SVM learning subsection of Methods requires clarification to distinguish which of these methods were developed in the previous Molecular Oncology publication and which were developed as part of this publications.*

 Response: This is now clarified within the first paragraph of the Methods section in the manuscript. The SVM classifier was adopted from previous Molecular Oncology publication, while the feature selection method has been developed as part of this publication.

*Comment 6: Details about the SVM learning algorithm are included in the caption to Figure 1, but must also be included and completely described in text for the corresponding section of the methods.*

 Response: Thanks for the reviewer’s suggestion. This description of the SVM learning algorithm has been moved from the Figure 1 legend and integrated into the first paragraph of the methods section.

*Comment 7: No equations are provided to describe the role of the parameters C and sigma. It is also unclear whether this greedy search is implemented by the Matlab function fitcsvm or uses custom code developed by the authors.*

 Response: A brief description of the role of each parameter has been added to the first paragraph of the methods section of the manuscript. Readers are also now directed to a reference (Ben-Hur and Weston, 2010) if more detail is desired.

 The greedy search, also called sequential backward feature selection, was implemented as a script by our lab in MATLAB. It is not a MATLAB function. This is clarified by changing a few words in the first paragraph of the methods section: “A backwards feature selection (greedy) algorithm was designed and implemented in MATLAB in which…”

 Moreover, as described above, the SVM classifier was adopted from previous Molecular Oncology publication (Dorman *et al.* 2016), while the feature selection method has been developed as part of this publication.

**Results**

*Comment 8: Need to specify whether reported accuracies are computed with leave-one-out cross validation or 9-fold cross validation (described in Methods).*

 Response: All SVM models described in the manuscript used leave-one-out cross validation except one, and this is clearly indicated in Table 1, and is now commented on in the methods. A 9-fold cross-validation was used to build a model using 735 patients who were treated with Chemotherapy and/or Hormone therapy, as leave-one-out cross validation of this many patients took an unreasonably long time to complete (it exceeded 3 weeks on a dedicated I7 Intel processor).

*Comment 9: Ideally, given the size of METABRIC data they would be calculated on independent training (first 1000 patient samples) and test (last 1000 patient samples) datasets.*

 Response: We obtained new results for both RF and mRMR+SVM models using Discovery patient set for training and Validation set for testing, however the performance of the model was poor. After further investigation, we found that there were large differences between gene expression levels of the 26 model signature genes in the Discovery versus Validation sets (we used Wilcoxon rank sum test, Kruskal-Wallis test and t-test to evaluate the results – shown in the plotted distributions of gene expression in Supplemental Dataset 2) regardless of patient status (alive or dead). Hence, building any classifier using discovery and validation set as training and test set in their current forms will result of poor performance due to this source of heterogeneity.

 To address this issue, we did carry out another experiment based on data from the Discovery patient dataset alone; using 70% of data for training and remaining 30% for testing, the performance of the model was significantly better. We speculate that the discrepancy between the expression distributions in the Discovery and Validation sets were the result of batch effects. The results have been added to the manuscript (Tables 4,5).

*Comment 10: AUC must be computed separately for discovery and validation sets (Table 2).*

 Response: We have included additional performance measures to Tables 1-5, including Area Under Curve (AUC).

*Comment 11: It is unclear whether the previous validation set described in the sentence “Predictions for the METABRIC patient cohort, which was independent of the previous validation set” refers to a validation set used in this publication or the previous publication.*

 Response: This sentence is referring to breast cancer patient data from Hatzis *et al.* (2013), which was used as a validation set in Dorman *et al. *(2016), not this publication. We have modified this sentence to clarify the issue.

*Comment 12: Covariates such as ER/PR or PAM50 subtypes must be included in a table describing the sample cohorts. Accuracy must be computed separately for these co-variates or they must also be included as co-variates in the machine-learning model.*

 Response: Even with the subtype as covariant, it is not possible to perform the analysis the reviewer requested. Certain therapies are definitely more effective in particular subtypes (eg. etoposide, docetaxel, and cisplatin are preferentially active in basal or claudin-low cell lines, as observed clinically; Heiser *et al., *2012). The public METABRIC dataset (or the corresponding publication) does not provide the specific therapies used to treat individual patients. Had they done so, it would have made sense to look at these covariates.

 Reference: Heiser LM, Sadanandam A, Kuo WL, Benz SC, Goldstein TC, Ng S, Gibb WJ, Wang NJ, Ziyad S, Tong F, *et al.* (2012). Subtype and pathway specific responses to anticancer compounds in breast cancer. *Proc Natl Acad Sci US A* **109**:2724-2729.

*Comment 13: Ideally accuracy would be compared to existing breast cancer classifiers (e.g., using code from Marchionni et al., BMC Genomics, 2013) and/or survival curves reported in the literature.*

 Response: The proposed method has been compared against the K-TSP (Marchionni *et al.*, BMC Genomics, 2013) as per reviewer’s suggestion and the results are presented in Table 6 of the manuscript.

**Conclusions**

*Comment 14: Must be discussed in the context of existing genomics classifiers for breast cancer (e.g., OncotypeDx and/or Mammaprint).*

 Response: We have added text to both the second paragraph of the “Results and Discussion” paragraph and to the conclusion of the paper.

*Comment 15: Results must be put in context with other predictions on METABRIC data, e.g., outcomes from the DREAM contest.*

 Response: An important distinction to note in regards to our methodology is that the predictions are based on the genes known to be associated with the response to specific drugs used to treat breast cancer. In the DREAM contest, the method with the highest METABRIC score (as described in Cheng *et al.*, 2013) was phenotype-based, finding signatures for molecular processes that are disregulated in METABRIC, rather than responses to the cancer therapies themselves. While this is an interesting prediction method, the results cannot compared to our approach. The gene signatures that we have derived contain components of many different pathways.

 Reference: Cheng WY, Ou Yang TH, Anastassiou D. Biomolecular events in cancer revealed by attractor metagenes. PLoS Comput Biol. 2013;9(2):e1002920.
